# Assessment of Factors Contributing to Health Outcomes in the Eight States of the Mississippi Delta Region

**DOI:** 10.5888/pcd13.150440

**Published:** 2016-03-03

**Authors:** Keith P. Gennuso, Amanda Jovaag, Bridget B. Catlin, Matthew Rodock, Hyojun Park

**Affiliations:** Author Affiliations: Amanda Jovaag, Bridget B. Catlin, Matthew Rodock, University of Wisconsin Population Health Institute, University of Wisconsin, Madison, Wisconsin; Hyojun Park, Department of Population Health Sciences, University of Wisconsin, Madison, Wisconsin.

## Abstract

**Introduction:**

The objective of this observational study was to examine the key contributors to health outcomes and to better understand the health disparities between Delta and non-Delta counties in 8 states in the Mississippi River Delta Region. We hypothesized that a unique set of contributors to health outcomes in the Delta counties could explain the disparities between Delta and non-Delta counties.

**Methods:**

Data were from the 2014 County Health Rankings for counties in 8 states (Alabama, Arkansas, Illinois, Kentucky, Louisiana, Mississippi, Missouri, and Tennessee). We used the Delta Regional Authority definition to identify the 252 Delta counties and 468 non-Delta counties or county equivalents. Information on health factors (eg, health behaviors, clinical care) and outcomes (eg, mortality) were derived from 38 measures from the 2014 County Health Rankings. The contributions of health factors to health outcomes in Delta and non-Delta counties were examined using path analysis.

**Results:**

We found similarities between Delta counties and non-Delta counties in the health factors (eg, tobacco use, diet and exercise) that significantly predicted the health outcomes of self-rated health and low birthweight. The most variation was seen in predictors of mortality; however, Delta counties shared 2 of the 3 significant predictors (ie, community safety and income) of mortality with non-Delta counties. On average across all measures, values in the Delta were 16% worse than in the non-Delta and 22% worse than in the rest of the United States.

**Conclusion:**

The health status of Delta counties is poorer than that of non-Delta counties because the health factors that contribute to health outcomes in the entire region are worse in the Delta counties, not because of a unique set of health predictors.

## Introduction

The Mississippi River Delta Region is among the most socioeconomically disadvantaged areas of the United States. The Delta is defined by the Delta Regional Authority as 252 counties or parishes in 8 states near the lower half of the Mississippi River (Figure). These counties typically have poorer health outcomes than peer counties in the same states and the rest of the country. Various measures of disease burden for Delta counties, such as mortality rates from all causes, cancer, and heart disease, are approximately 10% higher than for non-Delta counties in the same states (1) and 20% higher than the rates in the United States overall (2). Although mortality rates have decreased by 1% annually across the United States during the last 3 decades, the rate of decline has been much slower in the Delta, which had approximately 187 more deaths per 100,000 in 2004 than the rest of the country (2).

**Figure:**
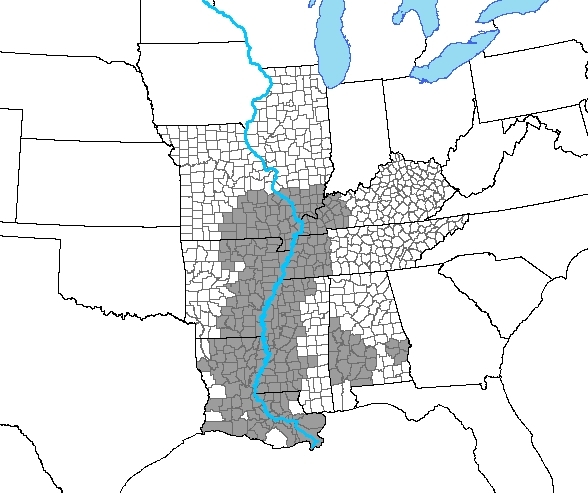
Delta counties (shaded [n = 252]) and non-Delta counties (n = 468) in the 8 states (Alabama, Arkansas, Illinois, Kentucky, Louisiana, Mississippi, Missouri, and Tennessee) that contain parts of the Mississippi River Delta Region.

Although several studies described the poor health status of the Mississippi River Delta Region, few used empirical methods to explain health disparities between the Delta and other regions. Bloom and Bowser (3) examined the contributions of income and nonincome factors to disparities in life expectancy between Delta counties and the rest of the United States across 30 years. They found that the contribution of income to the difference in life expectancy decreased from 64% in 1970 to 23% in 2000. Although that study’s findings are noteworthy because they provide empirical evidence of the importance of income to health in the Delta, more information is needed on the various nonincome factors that influence health outcomes in the region. Such studies require up-to-date, accurate, county-level health data. 

County Health Rankings (www.countyhealthrankings.org) — a collaborative effort between the University of Wisconsin Population Health Institute and the Robert Wood Johnson Foundation — compiles health-related information from various sources to produce annual rankings (4). We used county-level data from County Health Rankings for various health factors to identify the key contributors to health outcomes and to better understand the health disparities between Delta counties and non-Delta counties in the same states. We hypothesized that a unique set of contributors to health outcomes in the Delta counties could explain the disparity between Delta counties and non-Delta counties. An understanding of these contributors could assist community leaders and public health officials in the Delta in identifying strategies to improve the health of their communities.

## Methods

### Data and measures

Data for this study came from the 2014 County Health Rankings for counties in the 8 states — Alabama, Arkansas, Illinois, Kentucky, Louisiana, Mississippi, Missouri, and Tennessee — that make up the Mississippi River Delta Region (5). We used the Delta Regional Authority definition to identify the 252 Delta counties and parishes and the 468 non-Delta counties in the region. According to this definition, certain counties in Alabama, which is not proximal to the Mississippi River, are considered part of the Delta because they share socioeconomic indicators, political ideologies, and cultural commonalities. The conceptual model of population health used in this study was adapted from the County Health Rankings model (4). As in that model, health outcomes consisted of 3 measures: mortality, defined as years of potential life lost (YPLL) before the age of 75 years; self-rated health (self-report of fair or poor health); and low birth weight. Health factors consisted of 35 measures grouped into the following 5 broad categories: health behaviors, clinical care, social and economic factors, physical environment, and demographic variables. We adapted the County Health Rankings model to include demographic variables as the fifth category because we wanted to quantify the potential for race/ethnicity, sex, and the rurality of a county to influence health. Demographic variables are not included in the County Health Rankings model because that model’s focus is on modifiable, actionable contributors to health. 

### Analysis

To prepare data for the main analysis and put the measures on a standardized scale, *z* scores were calculated for each measure (excluding demographic variables) by using means and standard deviations derived from the 8 Delta states. Where data were missing, state-level mean values were imputed. We multiplied *z* scores for positive outcomes, such as high school graduation rate, by −1 so that all measures followed the same scheme, in which a higher number indicates poorer health. Composite scores were then calculated for 16 subcategories of health factors (Appendix), giving equal weight to each measure in a subcategory. For example, the health behavior subcategory of diet and exercise consisted of 4 measures, each given 25% weights.

For descriptive purposes, mean and standard deviation of raw values for each measure were calculated for Delta counties, non-Delta counties, and the 3,141 counties in the United States. Differences between groups for descriptive variables were examined by using 2-way analysis of variance with Tukey post-hoc testing. We used path analyses to examine the contributions of health factors to health outcomes in Delta and non-Delta counties. Because the purpose of this study was to assess the strength of these well established relationships and not to determine their existence, the path analysis model, based on the County Health Rankings model, was specified a priori, and no a posteriori model modification was performed. Direct paths were drawn from each of the 16 health factor subcategories to the 3 health outcomes. Standardized path coefficients were estimated to facilitate comparison across measures, and significance was determined as α = 0.05. Criteria used to examine the overall model fit were an incremental fit index (the Bentler Comparative Fit Index >.95), and two absolute fit indices (the root mean square error of approximation <.06 and the standardized root mean square residual <.08) (6). We calculated the amount of variance in health outcomes explained by the model as 1− disturbance (the path analysis equivalent to error in linear regression modeling). All analyses were performed in 2014 using SAS 9.4 (SAS Institute).

## Results

The analysis comparing Mississippi River Delta counties, non-Delta counties, and the national average on each of the 35 health factors and 3 health outcomes showed significant differences among the groups for most variables (Table 1). In general, values indicated poorer health in Delta counties compared with non-Delta counties and the national average except in drug and alcohol use and diabetic monitoring. For demographic variables, both Delta counties and non-Delta counties had Hispanic populations smaller than the national average. However, the Delta counties had a significantly smaller non-Hispanic white population and a larger African American population (nearly 3 times larger) compared with the national average. Non-Delta counties had more non-Hispanic whites and fewer African Americans compared with the national average.

**Table 1:** Descriptive Statistics for Delta Counties (n = 252) and Non-Delta Counties (n = 468) in Eight States in Mississippi River Delta Region and for US Counties Overall (n = 3,141), 2014^a^^,^^b^

Variable	Delta Counties	Non-Delta Counties	*P* Value^c^	National Average	*P* Value^d^
**Health outcomes**					
Mortality^e^, rate per 100,000	10,556 (2,023)	8,903 (2,129)	<.001	8,060 (2,407)	<.001
Self-rated fair or poor health	22.9 (4.6)	21.0 (6.8)	<.001	17.3 (6.1)	<.001
Low birth weight	10.8 (2.5)	8.7 (1.8)	<.001	8.3 (2.1)	<.001
**Health behaviors**					
Tobacco use	27.9 (2.6)	27.6 (3.4)	.41	24.8 (4.0)	<.001
Diet and exercise					
Access to exercise opportunities	39.1 (21.2)	47.4 (21.9)	<.001	52.3 (24.5)	<.001
Adult obesity	35.2 (3.6)	32.3 (2.8)	<.001	30.6 (4.2)	<.001
Food environment index^f^	6.34 (1.49)	7.52 (0.85)	<.001	7.37 (1.25)	<.001
Physical inactivity	33.3 (3.4)	31.5 (4.0)	<.001	27.9 (5.3)	<.001
Alcohol and drug use					
Excessive drinking	12.8 (3.8)	14.1 (5.2)	.05	16.5 (5.2)	<.001
Alcohol-impaired driving deaths	11.3 (12.8)	15.1 (28.2)	.34	17.4 (36.8)	.02
Sexual activity					
Sexually transmitted disease, rate per 100,000	612.28 (389.77)	337.54 (226.89)	<.001	354.63 (273.90)	<.001
Teen births, rate per 1,000	60.21 (16.45)	48.65 (15.13)	<.001	44.41 (20.03)	<.001
**Clinical care**					
Access to care					
Dentist, rate per 100,000	29.37 (16.30)	33.57 (18.17)	.07	39.28 (25.01)	<.001
Mental health provider, rate per 100,000	58.61 (65.57)	67.45 (69.61)	.50	87.87 (98.79)	<.001
Primary care physician, rate per 100,000	42.14 (24.09)	47.05 (25.93)	.14	55.24 (33.87)	<.001
Uninsured	19.2 (2.8)	17.4 (3.7)	<.001	18.0 (5.5)	<.001
Quality of care					
Diabetic monitoring	82.5 (4.5)	84.4 (4.5)	<.001	83.9 (6.7)	.002
Mammography screening	56.9 (6.8)	58.5 (7.4)	.03	60.8 (8.3)	<.001
Preventable hospital stays, rate per 1,000	99.23 (29.53)	94.85 (37.43)	.17	76.49 (29.88)	<.001
**Social and economic factors**					
Unemployment	9.2 (2.3)	8.3 (2.0)	<.001	7.7 (2.8)	<.001
Education					
High school graduation^g^	78.0 (9.5)	83.6 (7.0)	<.001	81.6 (9.7)	<.001
Some college	47.5 (9.2)	51.5 (10.7)	<.001	55.1 (11.8)	<.001
Income					
Median household income, $	36,652 (7,095)	41,358 (9,858)	<.001	44,829 (11,394)	<.001
Children in poverty	34.2 (9.3)	27.1 (8.4)	<.001	24.5 (9.2)	<.001
Family and social support					
Single-parent household	41.7 (12.5)	31.9 (7.8)	<.001	31.6 (10.3)	<.001
Inadequate social support	23.0 (5.2)	19.9 (5.1)	<.001	19.3 (5.4)	<.001
Community safety					
Injury deaths, rate per 100,000	87.06 (18.66)	82.02 (22.41)	.02	76.16 (24.64)	<.001
Violent crimes, rate per 100,000	390.31 (302.13)	255.32 (195.19)	<.001	257.12 (207.49)	<.001
**Physical environment**					
Air and water quality					
Air particulates^h^	12.22 (1.15)	12.24 (1.51)	.98	11.62 (1.53)	<.001
Water violations	11.3 (14.8)	8.6 (15.9)	.08	9.2 (16.8)	.13
Housing and transportation					
Driving alone to work	82.2 (3.9)	81.2 (4.1)	.16	78.2 (7.8)	<.001
Long commute driving alone	32.7 (10.7)	33.6 (11.0)	.57	29.6 (12.0)	<.001
Severe housing problems	14.6 (3.9)	13.0 (3.0)	<.001	14.1 (4.8)	.21
**Demographics**					
Female	50.5 (2.6)	50.5 (1.7)	.90	50.0 (2.2)	<.001
Race/ethnicity					
Non-Hispanic white	68.0 (22.8)	86.5 (12.6)	<.001	78.0 (19.9)	<.001
African American	27.7 (23.1)	7.5 (11.1)	<.001	8.8 (14.4)	<.001
Hispanic	2.4 (1.8)	3.5 (3.9)	.43	8.5 (13.3)	<.001
Rurality^i^	66.0 (26.9)	63.3 (28.9)	.50	58.6 (31.5)	<.001

a Values are mean (standard deviation) percentages unless otherwise noted.

b Data were from the 2014 County Health Rankings for counties in 8 states (Alabama, Arkansas, Illinois, Kentucky, Louisiana, Mississippi, Missouri, and Tennessee) (5).

c *P* value for the group differences between Delta counties and non-Delta counties.

d *P* value for the group differences between Delta counties and the national average.

e Defined as years of potential life lost (YPLL) before age 75, a measure of premature, or preventable, death.

f Values are an index of factors that contribute to a healthy food environment on a scale of 0 (worst) to 10 (best).

g Data for high school graduation rates are for 2010–2011 and do not include Idaho, Kentucky, or Oklahoma because these states had different methods of estimation than the rest of the country at that time.

h Values are average daily density of fine particulate matter less than 2.5 micrograms in diameter per cubic meter.

i Rurality is defined as the percentage of the population living in a rural area.

In the primary analysis, overall model fit was good in the sample of Delta counties: all 3 model-fit criteria were satisfied. Two of the 3 criteria were satisfied in the sample of non-Delta counties: the standardized root mean square residual and the Bentler Comparative Fit Index. The amount of variance in health outcomes explained by the model in the Delta counties was 67% of YPLL, 80% of low birth weight, and 34% of self-rated health; in the non-Delta counties variance was 78% of YPLL, 69% of low birth weight, and 59% of self-rated health (Table 2).

**Table 2:** Endogenous Variable Disturbance^a^, Health Outcomes in Counties of Eight States in the Mississippi River Delta Region, 2014

Variable	Estimate (SE)	*t* Value
**Delta counties**		
Mortality^b^	0.33 (0.04)	9.26
Self-rated fair or poor health	0.66 (0.05)	13.04
Low birth weight	0.20 (0.02)	8.48
**Non-Delta counties**		
Mortality^b^	0.22 (0.02)	10.86
Self-rated fair or poor health	0.41 (0.03)	12.49
Low birth weight	0.31 (0.03)	11.56

Abbreviation: SE, standard error.

a Similar to a residual in regression modeling: a disturbance represents the unexplained variance (or omitted causes) of an outcome variable.

b Defined as years of potential life lost (YPLL) before age 75, a measure of premature, or preventable, death.

Because higher *z* scores indicate worse health, a positive path coefficient for a nondemographic variable indicates a worsening of health outcomes with a worsening of health factors (Table 3). For demographic variables, a positive path coefficient indicates a worsening of health outcomes and a greater proportion of the population characterized by that variable. In the Delta counties, a few health factors stood out as significant predictors of health outcomes. Tobacco use (β = 0.40; standard error [SE], 0.11), diet and exercise (β = 0.38; SE, 0.11), and alcohol and drug use (β = −0.14; SE, 0.06) were the only significant predictors of self-rated health. Sexual activity (β = 0.18; SE, 0.07) and the percentage of the population that was African American (β = 0.64; SE, 0.10) were significant and the strongest predictors of low birth weight, followed by quality of care (β = 0.11; SE, 0.04) and the percentage of the population that was female (β = 0.11; SE, 0.04). The 3 significant predictors of YPLL were income (β = 0.26; SE, 0.08), the percentage of the population that was female (β = 0.23; SE, 0.05), and community safety (β = 0.17; SE, 0.05).

**Table 3:** Standardized Path Estimates^a^, Relationship Between Health Factors and Health Outcomes, Delta Counties and Non-Delta Counties in the Mississippi River Delta Region, 2014

Location/Health Factor	Fair or Poor Health, β (SE)	*t* Value	Low Birth Weight, β (SE)	*t* Value	YPLL, β (SE)	*t* Value
**Delta counties**						
Tobacco use	0.40 (0.11)	3.72	0.03 (0.06)	0.46	0.14 (0.08)	1.75
Diet and exercise	0.38 (0.11)	3.34	−0.05 (0.06)	−0.84	0.15 (0.08)	1.85
Alcohol and drug use	−0.14 (0.06)	−2.31	−0.04 (0.03)	−1.28	−0.02 (0.04)	−0.48
Sexual activity	−0.08 (0.12)	−0.66	0.18 (0.07)	2.83	0.10 (0.08)	1.17
Access to care	0.01 (0.08)	0.11	0.03 (0.05)	0.67	−0.01 (0.06)	−0.26
Quality of care	−0.03 (0.06)	−0.49	0.11 (0.04)	3.02	0.06 (0.05)	1.35
Education	−0.04 (0.09)	−0.48	0.08 (0.05)	1.56	0.02 (0.06)	0.32
Employment	0.01 (0.09)	0.09	−0.05 (0.05)	−1.09	0.01 (0.06)	0.20
Income	0.06 (0.11)	0.55	−0.03 (0.06)	−0.56	0.26 (0.08)	3.31
Family and social support	−0.06 (0.12)	−0.55	0.11 (0.06)	1.74	0.13 (0.08)	1.54
Community safety	−0.01 (0.07)	−0.14	0.02 (0.04)	0.64	0.17 (0.05)	3.67
Air and water	−0.06 (0.06)	−1.09	−0.02 (0.03)	−0.61	0.003 (0.04)	0.07
Housing and transportation	−0.08 (0.06)	−1.15	−0.03 (0.04)	−0.78	−0.05 (0.05)	−0.96
Female	0.003 (0.07)	0.05	0.11 (0.04)	2.77	0.23 (0.05)	4.69
Hispanic	−0.03 (0.07)	−0.40	0.01 (0.04)	0.33	−0.04 (0.05)	−0.79
Asian	0.12 (0.08)	1.39	−0.04 (0.05)	−0.86	0.01 (0.06)	0.22
African American	0.19 (0.17)	1.07	0.64 (0.10)	6.74	0.04 (0.12)	0.32
Rurality	0.11 (0.09)	1.26	0.02 (0.05)	0.39	0.09 (0.06)	1.39
**Non-Delta counties**						
Tobacco use	0.23 (0.08)	3.07	−0.05 (0.07)	−0.81	0.11 (0.06)	2.03
Diet and exercise	0.28 (0.06)	4.78	0.05 (0.05)	0.92	0.02 (0.04)	0.57
Alcohol and drug use	−0.08 (0.04)	−1.88	−0.13 (0.04)	−3.44	−0.10 (0.03)	−3.15
Sexual activity	0.01 (0.07)	0.11	0.03 (0.06)	0.55	0.10 (0.05)	1.98
Access to care	−0.12 (0.07)	−1.79	−0.04 (0.06)	−0.69	−0.13 (0.05)	−2.75
Quality of care	0.02 (0.05)	0.47	−0.03 (0.04)	−0.71	0.13 (0.03)	3.78
Education	−0.02 (0.06)	−0.31	0.14 (0.05)	2.73	0.01 (0.04)	0.32
Employment	0.05 (0.04)	1.15	0.03 (0.04)	0.94	−0.06 (0.03)	−2.16
Income	0.11 (0.07)	1.67	−0.09 (0.06)	−1.58	0.21 (0.05)	4.17
Family and social support	0.13 (0.06)	2.10	0.17 (0.05)	3.21	0.13 (0.04)	2.95
Community safety	0.10 (0.05)	2.20	0.13 (0.04)	3.21	0.31 (0.03)	9.09
Air and water	0.03 (0.04)	0.82	0.10 (0.03)	2.85	−0.002 (0.03)	−0.06
Housing and transportation	0.15 (0.04)	3.54	0.04 (0.04)	1.01	0.13 (0.03)	4.24
Female	0.03 (0.04)	0.72	0.02 (0.03)	0.52	0.06 (0.03)	2.21
Hispanic	0.10 (0.05)	1.99	−0.07 (0.04)	−1.63	−0.001 (0.04)	−0.01
Asian	0.07 (0.05)	1.31	0.03 (0.04)	0.59	0.01 (0.04)	0.28
African American	−0.07 (0.07)	−0.98	0.49 (0.06)	7.81	0.01 (0.05)	0.27
Rurality	0.24 (0.07)	3.60	0.10 (0.05)	1.71	0.26 (0.05)	5.15

a A positive path coefficient for a nondemographic variable indicates a worsening of health outcomes with a worsening of health factors. Higher values for nondemographic variables indicate poorer health; demographic variables are sex, race/ethnicity, and rurality.

The model for the non-Delta counties was characterized by a greater number of significant path coefficients. As in the Delta counties, diet and exercise (β = 0.28; SE, 0.06) and tobacco use (β = 0.23; SE, 0.08) were significant predictors of self-rated health; however, rurality was also a strong and significant predictor (β = 0.24; SE, 0.07). Percentage of the population that was African American was again the strongest significant predictor of low birth weight (β = 0.49; SE, 0.06), but family and social support (β = 0.17; SE, 0.05) proved to be significant and a stronger indicator than sexual activity (β = 0.03; SE, 0.06) in this sample. The top 3 significant predictors of YPLL in the non-Delta counties were community safety (β = 0.31; SE, 0.03), rurality (β = 0.26; SE, 0.05), and income (β = 0.21; SE, 0.05).

## Discussion

We used county-level data for a variety of health factors to identify the key contributors to health outcomes in the Mississippi River Delta Region and to better understand health disparities between Delta and non-Delta counties in the region’s states. Contrary to our hypothesis, our main finding was that the primary contributors to health outcomes in Delta and non-Delta counties were similar, especially for self-rated health and low birth weight. The predictors were most varied for mortality, but 2 of the 3 significant predictors in Delta counties were also the strongest significant predictors in non-Delta counties. Overall, the health status of Delta counties appeared to be poorer than that of non-Delta counties because the factors that affect health the most in the entire region were worse in the Delta counties, and not because there is an entirely different set of health predictors in Delta counties than in non-Delta counties.

These predictors also proved to be analogous to what was found by other studies examining predictors of similar health outcomes in a variety of populations. For instance, it is well understood that health behaviors are associated with self-rated health status. Two recent studies have presented evidence supporting the relationship between self-rated health and smoking, physical activity, and proper nutrition (7,8). Zarini et al (7) found a higher likelihood of reporting fair or poor self-rated health among people who consumed fewer vegetables and mostly high-fat foods in a sample of 1,701 US adults. In the same study, less physically active women were also significantly more likely to report fair or poor health. In a sample of nearly 4,000 elderly Russians, Selivanova and Cramm (8) found self-rated health to be significantly associated with physical activity among both sexes, smoking among men, and fruit and vegetable consumption among women.

Similarly, the link between African American race and low birth weight is well established. Women identifying as black or African American are twice as likely to have a preterm birth and 3 to 4 times as likely to have a very early preterm birth as other racial and ethnic groups in the United States and the United Kingdom (9,10). In the most recent Centers for Disease Control and Prevention data, low birth weight was the most common cause of death for African American infants, whereas congenital malformations were the leading cause for most other racial/ethnic groups (11). Theories tested to explain these wide racial/ethnic disparities include prenatal maternal behaviors, genetic disparities, social circumstances, and maternal stress; however, currently there is no agreement on why these racial/ethnic disparities exist (12).

Income is also a widely accepted predictor of mortality. In the United States, data from the National Longitudinal Mortality Study show that those in the highest income bracket (400% federal poverty level) can expect to live 6 years longer than those in the lowest bracket (≤100% federal poverty level) (13). Income inequality (14) and overall wealth (15) are also related to health. Hajat et al (15) found a significant trend of decreasing risk of mortality with increasing wealth, controlling for income and insurance, in a sample of approximately 21,000 US citizens. Those with a negative net worth had a 62% increased risk of mortality compared with those with a net worth of more than $500,000.

These findings suggest that the health disparities between Delta counties and non-Delta counties are related to differences in health factors that both areas have in common, rather than differences between the 2 areas in what affects health. For instance, for predictors of self-rated health status, we found no significant differences between Delta counties and non-Delta counties in tobacco use, but Delta counties scored worse in all 4 measures that make up the diet and exercise subcategory (ie, access to exercise opportunities, adult obesity, food environment index, and physical inactivity). Also, the African American proportion of the population, which was the strongest significant predictor of low birth weight, is roughly 4 times larger in Delta counties than non-Delta counties. Last, in regard to the primary contributors to our mortality measure, median household income is approximately 11% lower in Delta counties, and violent crime (one of 2 measures that make up community safety) is 53% higher.

Overall, Delta counties performed worse than non-Delta counties and the national average on all but 3 measures. On average across all measures, values in Delta counties were 16% worse than those in non-Delta counties and 22% worse than those in the rest of the United States. This trend is similar to what was previously reported in a small set of studies describing health in the Delta region (1,2). We found large differences (>20%) between Delta counties and the 2 other regions for the health outcomes of mortality and low birth weight and the health factors of sexually transmitted disease, teen births, single-parent households, and violent crime, among several others. The Delta counties performed better than the national average for excessive drinking and alcohol-impaired driving deaths and better than both non-Delta counties and the national average on diabetic monitoring.

The strengths and limitations of this study are congruous with those of our data source, the County Health Rankings. Rankings data are based on a commonly used and widely cited model of population health, and their annual report is one of the most complete sources of available data on population health measures for every county in the United States. Using the County Health Rankings model as the foundation of our models of path analysis was a strength of this study, and having access to the wide variety of data on health measures allowed us to examine the contributions of more health determinants than has previously been attempted in the Mississippi River Delta Region. However, we accept that there are limitations to these data as well. First, measures included in the County Health Rankings model are just one way in which the complex relationships between health factors and outcomes are hypothesized to exist. The model is based on publicly available, annually updated measures of county-level modifiable risk factors. Therefore, some relevant constructs, such as policies and programs implemented at the local, state, or federal levels, are omitted. Other models with different measures and alternative analyses are needed to gain a better understanding of the true relationship between health factors and outcomes. Another limitation stems from trying to predict current health outcomes with current health factors. The data for each year of County Health Rankings may have overlapped to some extent because some measures were aggregated for multiple years, and several health factor measures reflected later time periods than those of the health outcomes. Future research will benefit from more years of data for more measures to account for limitations associated with availability of current data, temporality, and reverse causality. Last, the classification of Delta counties by the Delta Regional Authority, whose goal is to foster regional economic development, is only one way to dichotomize counties in the 8 Delta states. However, it is the only widely accepted and used definition in the literature on the Mississippi River Delta Region. In addition, results from a sensitivity analysis we conducted provided evidence for a lack of differential classification by state, which also lends confidence to the use of this classification scheme.

Despite these limitations, this study contributes to the health disparities literature by examining contributors to health outcomes in the Mississippi River Delta Region. By examining the influence of 16 health factors comprising 35 measures on the health outcomes of mortality and quality of life, we saw that Delta counties and non-Delta counties were similar in regard to what contributes to health. Efforts to improve the health of Delta communities should focus on reducing the disparity in modifiable factors identified as predictors of health outcomes. For instance, our findings suggest that efforts to improve access to exercise opportunities (eg, the creation of parks and trails) and a healthy food environment (eg, fast-food limits, healthy food retailing), and subsequent reductions in obesity, could lead to improved health-related quality of life. In addition, taking action to reduce violent crime could help to address the disparity in premature mortality in the region. Resources, such as the County Health Rankings’ “What Works for Health” (http://www.countyhealthrankings.org/roadmaps/what-works-for-health) or the Centers for Disease Control and Prevention’s Healthy Living (http://www.cdc.gov/HealthyLiving/), which provide evidence ratings and implementation strategies for policies, programs, and system changes that improve health factors, can provide information on such efforts. We hope the findings of this study will assist local health officials, leaders, and policy makers in deciding how to allocate limited resources to improve the health of the Mississippi River Delta Region.

## Appendix 2014 County Health Rankings: Measures, Data Sources, and Years of Data

This file is available for download as a Microsoft Word document.

## References

[1] Felix H , Stewart MK . Health status in the Mississippi River Delta region. South Med J 2005;98(2):149–54. 10.1097/01.SMJ.0000145304.68009.02 15759943

[2] Cosby AG , Bowser DM . The health of the Delta Region: a story of increasing disparities. J Health Hum Serv Adm 2008;31(1):58–71. 18575148

[3] Bloom DE , Bowser DM . The population health and income nexus in the Mississippi River Delta Region and beyond. J Health Hum Serv Adm 2008;31(1):105–23. 18575150

[4] Remington PL , Catlin BB , Gennuso KP . The County Health Rankings: rationale and methods. Popul Health Metr 2015;13(1):11. 10.1186/s12963-015-0044-2 25931988 PMC4415342

[5] University of Wisconsin Population Health Institute. County health rankings and roadmaps. http://www.countyhealthrankings.org/rankings/data. Accessed August 8, 2014.

[6] Hu L , Bentler PM . Cutoff criteria for fit indexes in covariance structure analysis: conventional criteria versus new alternatives. Struct Equ Modeling 1999;6(1):1–55. 10.1080/10705519909540118

[7] Zarini GG , Vaccaro JA , Canossa Terris MA , Exebio JC , Tokayer L , Antwi J , Lifestyle behaviors and self-rated health: the living for health program. J Environ Public Health 2014;2014:315042. 10.1155/2014/315042 25530764 PMC4228703

[8] Selivanova A , Cramm JM . The relationship between healthy behaviors and health outcomes among older adults in Russia. BMC Public Health 2014;14(1):1183. 10.1186/1471-2458-14-1183 25410349 PMC4251861

[9] Goldenberg RL , Culhane JF , Iams JD , Romero R . Epidemiology and causes of preterm birth. Lancet 2008;371(9606):75–84. 10.1016/S0140-6736(08)60074-4 18177778 PMC7134569

[10] Martin JA , Hamilton BE , Osterman MJ , Curtin SC , Matthews TJ . Births: final data for 2013. Natl Vital Stat Rep 2015;64(1):1–65. 25603115

[11] Mathews TJ , MacDorman MF . Infant mortality statistics from the 2009 period linked birth/infant death data set. Natl Vital Stat Rep 2013;61(8):1–27. 24979974

[12] Bryant AS , Worjoloh A , Caughey AB , Washington AE . Racial/ethnic disparities in obstetric outcomes and care: prevalence and determinants. Am J Obstet Gynecol 2010;202(4):335–43. 10.1016/j.ajog.2009.10.864 20060513 PMC2847630

[13] Braveman PA , Cubbin C , Egerter S , Williams DR , Pamuk E . Socioeconomic disparities in health in the United States: what the patterns tell us. Am J Public Health 2010;100(Suppl 1):S186–96. 10.2105/AJPH.2009.166082 20147693 PMC2837459

[14] Pickett KE , Wilkinson RG . Income inequality and health: a causal review. Soc Sci Med 2015;128:316–26. 10.1016/j.socscimed.2014.12.031 25577953

[15] Hajat A , Kaufman JS , Rose KM , Siddiqi A , Thomas JC . Long-term effects of wealth on mortality and self-rated health status. Am J Epidemiol 2011;173(2):192–200. 10.1093/aje/kwq348 21059808 PMC3139960

