# Supplementary material for: Assessment of Factors Contributing to Health Outcomes in the Eight States of the Mississippi Delta Region
Source: Prev Chronic Dis. 2016 Mar 3;13:E33. doi: 10.5888/pcd13.150440 (PMC4778371; doi:10.5888/pcd13.150440)
Supplement: Supplementary file 1 [file 15_0440_Appendix.docx]

Appendix. 2014 County Health Rankings: Measures, Data Sources, and Years of Data

| **Variable** | **Measure** | **Data Source** | **Years of Data** |
| --- | --- | --- | --- |
| **Health outcomes** | | | |
| Length of life | Premature death | National Center for Health Statistics | 2008–2010 |
| Quality of life | Self-rated poor or fair health | Behavioral Risk Factor Surveillance System | 2006–2012 |
|  | Low birth weight | National Center for Health Statistics | 2005–2011 |
| **Health factors** | | | |
| **Health behaviors** | | | |
| Tobacco use | Adult smoking | Behavioral Risk Factor Surveillance System | 2012 |
| Diet and exercise | Adult obesity | NCCDPHP | 2010 |
|  | Food environment index^a^ | US Department of Agriculture Food Environment Atlas, Map the Meal Gap | 2010–2011 |
|  | Physical inactivity | NCCDPHP | 2010 |
|  | Access to exercise opportunities^a^ | OneSource Global Business Browser, Delorme map data, Esri, and US Census Tigerline Files | 2010, 2012 |
| Alcohol and drug use | Excessive drinking | Behavioral Risk Factor Surveillance System | 2006–2012 |
|  | Alcohol-impaired driving deaths | Fatality Analysis Reporting System | 2008–2012 |
| Sexual activity | Sexually transmitted infections | National Center for HIV/AIDS, Viral Hepatitis, STD, and TB prevention | 2011 |
|  | Teen births | National Center for Health Statistics | 2005–2011 |
| Clinical care | | | |
| Access to care | Uninsured | Small Area Health Insurance Estimates | 2011 |
|  | Primary care physicians | HRSA Area Resource File | 2011 |
|  | Dentists | HRSA Area Resource File | 2012 |
|  | Mental health providers | Centers for Medicare and Medicaid Services, National Provider Identification File | 2013 |
| Quality of care | Preventable hospital stays | Medicare/Dartmouth Institute | 2011 |
|  | Diabetic monitoring^a^ | Medicare/Dartmouth Institute | 2011 |
|  | Mammography screening^a^ | Medicare/Dartmouth Institute | 2011 |
| Social and economic factors | | | |
| Education | High school graduate^a^ | Data.gov, supplemented with National Center for Education Statistics | 2010–2011 |
|  | Some college^a^ | American Community Survey | 2008–2012 |
| Employment | Unemployment | Bureau of Labor Statistics | 2012 |
| Income | Household income^a^ | Small Area Income and Poverty Estimates | 2012 |
|  | Children in poverty | American Community Survey | 2012 |
| Family and social support | Inadequate social support | Behavioral Risk Factor Surveillance System | 2005–2010 |
|  | Children in single-parent households | American Community Survey | 2008–2012 |
| Community safety | Violent crime | Uniform Crime Reporting, Federal Bureau of Investigation | 2009–2011 |
|  | Injury deaths | CDC WONDER | 2006–2010 |
| Physical environment | | | |
| Air and water quality | Air pollution - particulate matter | CDC WONDER | 2011 |
|  | Drinking water violations | Safe Drinking Water Information System | 2012 –2013 |
| Housing and transportation | Severe housing problems | HUD, Comprehensive Housing Affordability Strategy | 2006–2010 |
|  | Driving alone to work | American Community Survey | 2008–2012 |
|  | Driving alone for long commute | American Community Survey | 2008–2012 |
| Demographic variables | | | |
| Female | Female population | US Census Bureau | 2012 |
| Race/ethnicity | Non-Hispanic white | US Census Bureau | 2012 |
|  | African American | US Census Bureau | 2012 |
|  | Hispanic | US Census Bureau | 2012 |
| Rurality^b^ | Percentage rural | 2010 Decennial Census | 2010 |

Abbreviations: CDC WONDER, Centers for Disease Control and Prevention Wide-ranging Online Data for Epidemiologic Research; Esri, Environmental Systems Research Institute; HRSA, Health Resources and Services Administration; HUD, US Department of Housing and Urban Development; NCCDPHP, National Center for Chronic Disease Prevention and Health Promotion.

^a^ Reverse-coded measures.

^b^ Rurality is defined as the percentage of the population living in a rural area.
